# Correction: Liu et al. Apigenin Ameliorates Hyperuricemia and Renal Injury through Regulation of Uric Acid Metabolism and JAK2/STAT3 Signaling Pathway. *Pharmaceuticals* 2022, *15*, 1442

**DOI:** 10.3390/ph16060819

**Published:** 2023-05-31

**Authors:** Tianyuan Liu, Huimin Gao, Yueyi Zhang, Shan Wang, Meixi Lu, Xuan Dai, Yage Liu, Hanfen Shi, Tianshu Xu, Jiyuan Yin, Sihua Gao, Lili Wang, Dongwei Zhang

**Affiliations:** 1 Diabetes Research Center, Traditional Chinese Medicine School, Beijing University of Chinese Medicine, Beijing 100029, China; 2 Institute of Chinese Materia Medica, China Academy of Chinese Medical Science, Beijing 100700, China; 3 Department of TCM Pharmacology, Chinese Material Medica School, Beijing University of Chinese Medicine, Beijing 102488, China

## Error in Figure

In the original publication [1], there were mistakes in Figures 5 and 9 as published. In Figure 5C, the molecular weight of URAT1 protein was labeled incorrectly. The correct size of URAT1 protein in this experiment should be 46 KDa. In Figure 9, the dosage of hypoxanthine (Hypox) was incorrectly stated in the original publication. The dosage of Hypox should be 500 mg/kg. The corrected Figure 5 and Figure 9 appear below.

## Text Correction

There was an error in the original publication. The dosage of hypoxanthine (Hypox) was incorrectly stated in the original publication. The dosage of Hypox should be 500 mg/kg.

A correction has been made to **Section 3. Discussion**, **Paragraph 8**:

“In this study, Allop was demonstrated to decrease the levels of liver XOD and serum UA, and increase the levels of urine CRE (day 6) in this rapid HUA mouse model. However, Allop did not reverse the increased serum levels of CRE and BUN in HUA mice. By contrast, Chen et al. [64] reported that Allop was able to decrease serum levels of CRE and BUN in HUA mice. The underlying cause to explain the conflicting findings may be attributed to the differences in the approaches to establish HUA mouse model. In their study, the authors employed intraperitoneal injection with PO to establish the HUA mouse model. However, in the present study, Hypox (500 mg/kg, gavage) was used to induce HUA mice in addition to PO exposure.”

The authors state that the scientific conclusions are unaffected. This correction was approved by the Academic Editor. The original publication has also been updated.

## Figures and Tables

**Figure 5:**
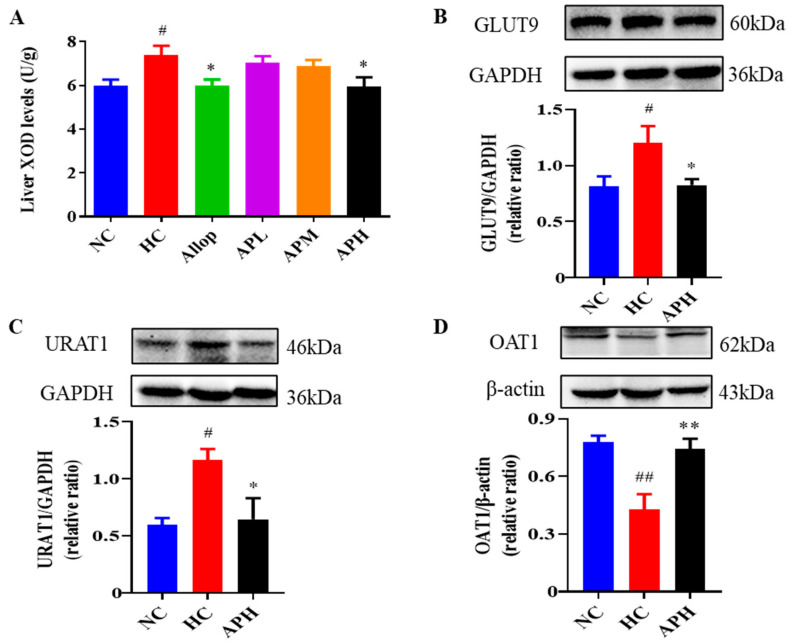
Apigenin decreases liver XOD levels (**A**; U/g, *n* = 10) and renal expression levels of GLUT9 and URAT1, and increases renal expression levels of OAT1 in HUA mice. Representative images of western blot and their analyses show GLUT9 (**B**), URAT1 (**C**), and OAT1 (**D**) expressions in the kidneys (*n* ≥ 3). ^##^ *p* < 0.01, ^#^ *p* < 0.05 vs. the NC group. ** *p* < 0.01, * *p* < 0.05 vs. the HC group.

**Figure 9:**
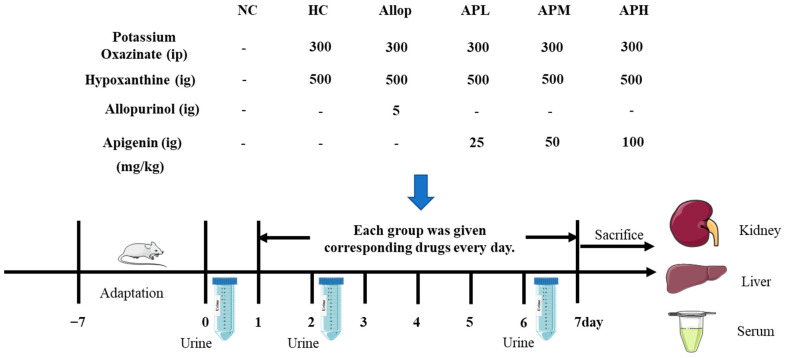
Experimental design on action of apigenin on acute HUA mice. ig denotes intragastrical administration, ip denotes intraperitoneal administration.
